# Ovarian Torsion and Its Remediation in a Three-Year-Old Girl

**DOI:** 10.7759/cureus.32132

**Published:** 2022-12-02

**Authors:** Megan S Coble, Amanda Schoonover, Christopher Benner, Todd Chassee

**Affiliations:** 1 Pediatric Emergency Medicine, Michigan State University College of Human Medicine, Grand Rapids, USA; 2 Medicine, Michigan State University College of Human Medicine, Grand Rapids, USA; 3 Emergency Medicine, Helen DeVos Children’s Hospital - Spectrum Health Medical Group, Grand Rapids, USA; 4 Emergency Medicine, Spectrum Health Medical Group, Grand Rapids, USA

**Keywords:** pediatric, doppler ultrasound, ovarian detorsion, case report, ovarian torsion

## Abstract

Ovarian torsion is a rare, emergent occurrence seen in the premenarchal population. If detected promptly, ovarian torsion can be treated via detorsion. We present a case of a three-year-old girl whose ovary spontaneously torsed and was corrected via ovarian detorsion. The patient presented with sudden-onset abdominal pain and emesis; a transabdominal ultrasound with Doppler was performed, which led to the diagnosis of ovarian torsion. The patient was directly taken into surgery for correction, after which she quickly recovered and was subsequently discharged. The choice of ovarian detorsion to protect fertility in pediatric patients is supported by this case and by the related literature. The key to safeguarding fertility in these patients lies in rapid detection, which remains a challenge in the pediatric population. By raising widespread awareness of the use of Doppler ultrasound as well as symptom presentation, the protection of fertility in cases of pediatric ovarian torsion can be improved.

## Introduction

Ovarian torsion is an emergent condition that is very rarely seen in the premenarchal population [1]. It is characterized by the twisting of the ovary and fallopian tube around the infundibulopelvic ligament and the ovarian ligament. This leads to the restriction of flow from the ovarian arteries, veins, and/or lymphatic ducts, which are contained within these two ligaments. This lack of blood flow places the ovary in a state of ischemia [2]. The largest risk factor for ovarian torsion is an ovarian mass or any other factor that increases the size of the ovary. Pediatric patients typically have normal ovaries with less likelihood of mass, making torsion much less common, especially before the onset of menses [2]. This case report describes the presentation, treatment, and recovery of a three-year-old female with ovarian torsion, a unique finding due to the patient’s young age.

## Case presentation

A three-year-old female patient presented to the emergency department of a tertiary care pediatric hospital with severe abdominal pain that had started less than 24 hours prior. The patient had experienced sudden onset of emesis and dysuria accompanying the pain in the early morning. She was neither febrile nor did she have diarrhea. She had been diagnosed with a urinary tract infection one month prior via urinalysis with subsequent mixed flora in the urine culture. She had no other significant medical, family, or social history. The history of the present illness was associated with emesis that had started abruptly that morning with no associated bowel distress. The patient was slightly tachypneic on admission to the emergency room, but her vital signs were otherwise within the normal range. She had a rectal temperature of 36.4 °C, heart rate of 110 beats per minute, respiratory rate of 32 breaths per minute, blood pressure of 102/42 mmHg, and oxygen saturation of 100%. The physical exam found periumbilical tenderness. The patient appeared uncomfortable during the exam and kept pushing the physician’s hand away during the abdominal examination. The abdomen was soft with no distension, and bowel sounds were normal. No costovertebral angle tenderness was detected. The rest of the examination was not significant.

**Figure 1:**
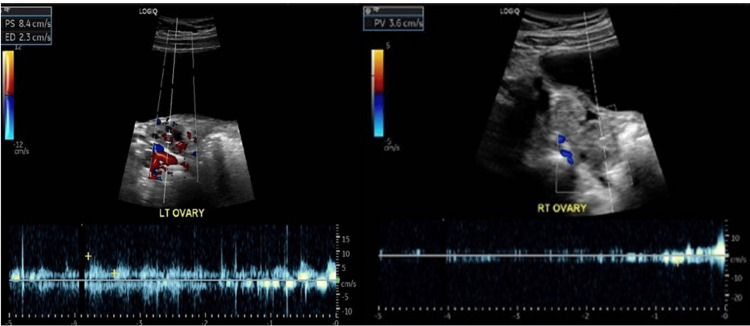
Doppler ultrasound of patient's ovaries The images demonstrate normal blood flow to the patient's left ovary (left) compared to the restricted blood flow to the torsed right ovary (right)

A urinalysis with microscopy was performed and was negative for glucose, significant red/white cells, and nitrite. There were trace ketones and slight protein. An ultrasound of the appendix was performed, eliciting no abnormal appendiceal findings. There were no gallbladder stones demonstrated. Due to pain in the right lower quadrant, and a grade one collection dilation of the right kidney seen on the ultrasound, a transabdominal ultrasound with Doppler was performed with emphasis on the right lower quadrant where the pain was most severe. Mild hydronephrosis was found to be creating pressure on the right ureter, leading to Doppler being performed. The ultrasound detected an enlarged right ovary with potential torsion. The left ovary measured 2.5 x 1.0 x 1.7 cm in dimension with a volume of 2.1 mL, while the right ovary measured 3.9 x 1.9 x 3.4 cm in dimension with a volume of 13.4 mL. Blood flow to the left ovary was intact (Figure 1), but that to the right ovary was limited to peripheral flow (Figure 1), suggestive of right ovarian torsion. There was no report of free fluid in the pelvis. The ultrasound was otherwise unremarkable with no findings pertaining to the appendix, uterus, or endometrium.

Based on the ultrasound findings, the patient was referred immediately to the pediatric surgical team for a consultation. Laparoscopic ovarian detorsion with hemorrhagic cyst aspiration was performed the same evening. When originally viewed via laparoscopy, the ovary contained a hemorrhagic cyst and appeared dusky. Following detorsion and aspiration of the cyst, the tissue appeared hemostatic and viable. Oophoropexy was not performed. The surgery was uncomplicated, and the patient tolerated the procedure well. She recovered quickly on the floor and was subsequently discharged in stable condition. However, she required acetaminophen and ibuprofen postoperatively for pain control. A follow-up pelvic ultrasound was ordered at two weeks post-discharge to ensure proper healing and to evaluate for recurrent ovarian torsion.

## Discussion

Ovarian torsion is a gynecological emergency that affects a large number of women every year. A retrospective study that reviewed surgical emergencies over a 10-year period in a women’s hospital found that ovarian torsion was the fifth leading cause of surgical intervention; of those, only 20% of cases involved premenarchal girls [1-3]. Of note, 71% of all cases of ovarian torsion occur in patients over 20 years of age [1]. Within the pediatric population, 52% of all cases occur in children aged 9-14 years [2]. Despite its rarity, ovarian torsion accounts for up to 2.7% of all cases of acute abdominal pain, with an incidence rate ranging from 0.3% to 3.5% of cases per year [4]. Ovarian torsion in patients who are not menarchal or postmenarchal appears to be exceedingly rare, especially in the absence of sizeable ovarian or pelvic masses.

In cases of ovarian torsion in pediatric patients with normal-sized ovaries with no ovarian mass, the etiology is not well understood [2]. Many underlying pathophysiological manifestations have been suggested to explain this phenomenon. One theory pertains to the disproportionate size of the uterus and the utero-ovarian ligaments. In the pediatric population, the uterus is relatively small in comparison with the utero-ovarian ligaments which are proportionally long. This large size difference accounts for the higher mobility of the ovaries resulting in a higher chance of the ovary twisting upon itself [2]. More commonly, there is an underlying pathological cause for torsion. These pathologies can include but are not limited to cystic teratomas or dermoids (31%), follicular or hemorrhagic ovarian cysts (23%-33%), and paraovarian/paratubal cysts, cystadenomas, or hydrosalpinx [2].

Despite the pathological findings that may accompany ovarian torsion in the pediatric population, cases involving normal-sized ovaries are not uncommon in premenarchal girls [2]. Of the pediatric cases that do present with masses, the majority of these masses are benign. A study conducted in 2010 reviewed 66 cases of patients with ovarian torsion. Of those cases, two (3.0%) had malignant growths, eight (12.1%) cases were normal with no growth, and 56 (84.9%) cases had benign growths [5]. This study is not unique, as the literature overwhelmingly shows that a majority of cases of pediatric ovarian torsion are accompanied by benign lesions.

Treatment of ovarian torsion primarily aims at protecting fertility as the vast majority of pediatric cases occur without malignant etiology [3]. A literature review found that an average of 1.8% of pediatric ovarian torsion cases were associated with malignancy, further indicating that fertility-sparing detorsion rather than resection should be the preferred option [6]. As is the case with this pediatric patient, if the ovary can be spared, it is the best option to help preserve fertility. Procedural options for ovarian torsion include ovarian detorsion, ovarian cystectomy, and oophoropexy with oophorectomy reserved as a last resort [3].

Another layer of complexity lies in the debate surrounding whether oophoropexy is beneficial in conjunction with ovarian detorsion. As with this case, oophoropexy is not routinely performed with ovarian detorsion, even though support for this combination is becoming increasingly popular. Retorsion following the detorsion of an ovary in the pediatric population is very risky, with retorsion rates currently ranging from 5% to 18% [7]. Oophoropexy is a surgical procedure that has been proposed to be performed in conjunction with oophorectomy to prevent retorsion. There are two methods of oophoropexy: plication of the adnexal ligaments or fixation of the adnexa to the pelvic side wall. A thorough review of the existing literature pertaining to oophoropexy in cases of pediatric ovarian torsion did not demonstrate any clear evidence supporting oophoropexy in the first instance of ovarian torsion [8]. There is currently much conflicting evidence: while case reports of successful oophoropexy have been published, there have also been reports of retorsion in pediatric patients despite oophoropexy [9]. Though there is little evidence, it has also been proposed that the alteration in anatomy due to oophoropexy may lead to decreased fertility [10]. Presently, the determination of whether oophoropexy should be conducted is a decision that is made on a case-by-case basis until more research on the topic is available [8].

The main challenge in diagnosing pediatric patients with ovarian torsions is its rarity, especially before the onset of menses. In addition, the benign nature of many cases and the broad, non-specific symptoms make the diagnosis difficult [11]. As is the case with younger patients, the description of symptoms within the pediatric community can prove to be a challenging part of forming a differential diagnosis. Ovarian torsion is no exception. Several studies have found that the symptom most associated with ovarian torsion is abdominal pain [12-14]. Another study has found that this abdominal pain does not have to be isolated to a particular unilateral lower quadrant in cases of ovarian torsion [14]. Table 1 outlines the wide variety of abdominal pain characteristics seen in pediatric ovarian torsion cases in one retrospective review [12]. 

**Table 1:** Abdominal pain characteristics in pediatric ovarian torsion cases* *[12]

Characteristic	Number of cases (%)
Severity	
Mild	4 (6)
Moderate	16 (23)
Severe	48 (69)
Quality	
Intermittent	43 (57)
Constant	32 (43)
Location	
Right lower quadrant	43 (61)
Left lower quadrant	24 (34)
Suprapubic	4 (6)
Physical exam characteristics	
Abdominal tenderness	72 (91)
Guarding	19 (27)
Rebound	11 (18)
Distention	4 (6)

Despite the difficulty in diagnosing ovarian torsion based on history alone, timely treatment is vital in ensuring that the ovary is salvageable. A medical case review found that regardless of the duration of symptoms prior to the examination, prompt operative intervention is essential in salvaging ovaries in the case of ovarian torsion. The authors reported that patients receiving operative intervention within the first eight hours of initial examination had a salvage rate of 40%. At 24 hours, the rate dropped to 33%, and after 24 hours from the initial exam, the rate was 0% [15]. Though these findings pertain to an adult population, similar results have been extrapolated to pediatric patients.

Diagnosing ovarian torsion relies on the use of prompt imaging, particularly ultrasound. Ultrasound has many benefits when used as a diagnostic tool, including its wide availability, lack of radiation, and rapid results [16]. It is important to note that ovarian torsion on ultrasound may present differently in the pediatric population compared to cases in adults. Though it was utilized in this case, one such limitation involves the use of color Doppler flow. Though color Doppler can be a useful tool, it is important to note that Doppler ultrasound may show normal findings in pediatric ovarian torsion, especially in the early stages of presentation [16]. One study has listed common potential image findings of ovarian torsion within the pediatric population, which are as follows: unilateral ovarian enlargement, atypical ovarian positioning, free fluid in the pelvis, and adnexal masses or cysts [16]. A meta-analysis has found an overall sensitivity of 92% and a specificity of 96% for the detection of ovarian torsion in pediatrics. This study also indicated that CT has very low sensitivity, making this a poor diagnostic tool for ovarian torsion [17-18]. 

Ovarian torsion in pediatric patients has an overall positive prognosis if treated promptly and adequately. The main challenge in making the diagnosis is to consider it in the initial differential, given its rarity in the first decade of life. The diagnosis should be considered in all pediatric patients presenting with abdominal pain, particularly those with sudden onset or unilateral pain. The presentation may be vague, and hence it is important to ensure that it is routinely considered in female pediatric abdominal pain presentations. Once considered in the diagnosis, ultrasound should be used to further investigate the condition. When utilizing ultrasound, it is critical to be aware that blood flow may be normal on Doppler, especially in the early stages of presentation. If ovarian torsion is suspected, consultation with the appropriate surgical subspecialty is mandatory.

## Conclusions

Ovarian torsion is a rare finding in pediatric patients. The underlying mechanism behind ovarian torsion in the pediatric population is not completely understood, especially in cases without ovarian or pelvic masses. Ovarian ultrasound with Doppler is the test of choice for diagnosis, even though normal or symmetric blood flow on Doppler does not rule out the possibility of ovarian torsion. As demonstrated in this case, ovarian detorsion is the best choice for pediatric patients without an ovarian/pelvic mass as this procedure protects fertility.

## References

[1] Guile SL, Mathai JK (2022). Ovarian Torsion. http://www.ncbi.nlm.nih.gov/books/NBK560675/.

[2] Poonai N, Poonai C, Lim R, Lynch T (2013). Pediatric ovarian torsion: case series and review of the literature. Can J Surg.

[3] Huang C, Hong MK, Ding DC (2017). A review of ovary torsion. Ci Ji Yi Xue Za Zhi.

[4] Adeyemi-Fowode O, Lin EG, Syed F, Sangi-Haghpeykar H, Zhu H, Dietrich JE (2019). Adnexal torsion in children and adolescents: a retrospective review of 245 cases at a single institution. J Pediatr Adolesc Gynecol.

[5] Tielli A, Scala A, Alison M, Vo Chieu VD, Farkas N, Titomanlio L, Lenglart L (2022). Ovarian torsion: diagnosis, surgery, and fertility preservation in the pediatric population. Eur J Pediatr.

[6] Wang JH, Wu DH, Jin H, Wu YZ (2010). Predominant etiology of adnexal torsion and ovarian outcome after detorsion in premenarchal girls. Eur J Pediatr Surg.

[7] Melcer Y, Sarig-Meth T, Maymon R, Pansky M, Vaknin Z, Smorgick N (2016). Similar but different: a comparison of adnexal torsion in pediatric, adolescent, and pregnant and reproductive-age women. J Womens Health (Larchmt).

[8] Dasgupta R, Renaud E, Goldin AB (2018). Ovarian torsion in pediatric and adolescent patients: a systematic review. J Pediatr Surg.

[9] Kurtoglu E, Kokcu A, Danaci M (2014). Asynchronous bilateral ovarian torsion. A case report and mini review. J Pediatr Adolesc Gynecol.

[10] Spinelli C, Buti I, Pucci V (2013). Adnexal torsion in children and adolescents: new trends to conservative surgical approach -- our experience and review of literature. Gynecol Endocrinol.

[11] Karaman E, Beger B, Çetin O, Melek M, Karaman Y (2017). Ovarian torsion in the normal ovary: a diagnostic challenge in postmenarchal adolescent girls in the emergency department. Med Sci Monit.

[12] Oltmann SC, Fischer A, Barber R, Huang R, Hicks B, Garcia N (2010). Pediatric ovarian malignancy presenting as ovarian torsion: incidence and relevance. J Pediatr Surg.

[13] Rousseau V, Massicot R, Darwish AA, Sauvat F, Emond S, Thibaud E, Nihoul-Fékété C (2008). Emergency management and conservative surgery of ovarian torsion in children: a report of 40 cases. J Pediatr Adolesc Gynecol.

[14] Rossi BV, Ference EH, Zurakowski D, Scholz S, Feins NR, Chow JS, Laufer MR (2012). The clinical presentation and surgical management of adnexal torsion in the pediatric and adolescent population. J Pediatr Adolesc Gynecol.

[15] Anders JF, Powell EC (2005). Urgency of evaluation and outcome of acute ovarian torsion in pediatric patients. Arch Pediatr Adolesc Med.

[16] Schuh AM, Klein EJ, Allred RJ, Christensen A, Brown JC (2017). Pediatric adnexal torsion: not just a postmenarchal problem. J Emerg Med.

[17] Sintim-Damoa A, Majmudar AS, Cohen HL, Parvey LS (2017). Pediatric ovarian torsion: spectrum of imaging findings. Radiographics.

[18] Bronstein ME, Pandya S, Snyder CW, Shi Q, Muensterer OJ (2015). A meta-analysis of B-mode ultrasound, Doppler ultrasound, and computed tomography to diagnose pediatric ovarian torsion. Eur J Pediatr Surg.

